# {2,2′-[(2,2-Dimethylpropane-1,3-diyl­dinitrilo)bis(phenylmethylidyne)]di­phenolato}copper(II)

**DOI:** 10.1107/S1600536811028844

**Published:** 2011-07-23

**Authors:** Hadi Kargar, Reza Kia, Majid Moghadam, Fatemeh Froozandeh, Muhammad Nawaz Tahir

**Affiliations:** a Chemistry Department, Payame Noor University, Tehran 19395-4697, Iran; b X-ray Crystallography Laboratory, Plasma Physics Research Center, Science and Research Branch, Islamic Azad University, Tehran, Iran; c Department of Chemistry, Science and Research Branch, Islamic Azad University, Tehran, Iran; d Catalysis Division, Department of Chemistry, University of Isfahan, Isfahan 81746-73441, Iran; e Department of Physics, University of Sargodha, Punjab, Pakistan

## Abstract

The complete mol­ecule of the title complex, [Cu(C_31_H_28_N_2_O_2_)], is generated by the application of twofold symmetry; the Cu and CMe_2_ atoms lie on the axis. The geometry around the Cu^II^ atom is distorted square-planar. The dihedral angle between the two phenyl rings is 76.0 (3) °. The crystal packing is stabilized by inter­molecular C—H⋯π inter­actions.

## Related literature

For background to tetra­dentate Schiff bases and their complexes, see, for example: Kargar *et al.* (2009 ▶, 2010 ▶).
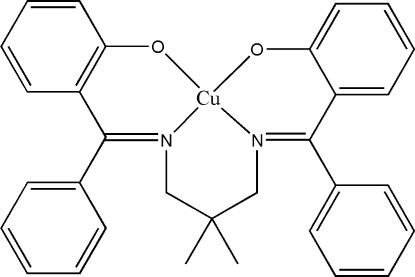


## Experimental

### 

#### Crystal data

[Cu(C_31_H_28_N_2_O_2_)]
*M* *_r_* = 524.09
Tetragonal, 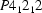

*a* = 9.7435 (14) Å
*c* = 25.717 (6) Å
*V* = 2441.5 (8) Å^3^
*Z* = 4
Mo *K*α radiation
μ = 0.93 mm^−1^
*T* = 291 K
0.21 × 0.11 × 0.08 mm

#### Data collection

STOE IPDS 2T Image Plate diffractometer
Absorption correction: multi-scan [*MULABS* (Blessing, 1995 ▶) in *PLATON* (Spek, 2009 ▶)] *T*_min_ = 0.995, *T*_max_ = 1.000
5595 measured reflections
2376 independent reflections
1380 reflections with *I* > 2σ(*I*)
*R*_int_ = 0.088

#### Refinement

*R*[*F* ^2^ > 2σ(*F* ^2^)] = 0.070
*wR*(*F* ^2^) = 0.064
*S* = 0.84
2376 reflections
165 parameters
H-atom parameters constrained
Δρ_max_ = 0.83 e Å^−3^
Δρ_min_ = −0.47 e Å^−3^
Absolute structure: Flack (1983 ▶), 918 Friedel pairs
Flack parameter: 0.00 (3)

### 

Data collection: *X-AREA* (Stoe & Cie, 2009 ▶); cell refinement: *X-AREA*; data reduction: *X-AREA*; program(s) used to solve structure: *SHELXTL* (Sheldrick, 2008 ▶); program(s) used to refine structure: *SHELXTL*; molecular graphics: *SHELXTL*; software used to prepare material for publication: *SHELXTL* and *PLATON* (Spek, 2009 ▶).

## Supplementary Material

Crystal structure: contains datablock(s) global, I. DOI: 10.1107/S1600536811028844/tk2765sup1.cif

Structure factors: contains datablock(s) I. DOI: 10.1107/S1600536811028844/tk2765Isup2.hkl

Additional supplementary materials:  crystallographic information; 3D view; checkCIF report

## Figures and Tables

**Table:** 

Cu1—O1	1.891 (3)
Cu1—N1	1.966 (4)

**Table:** 

O1—Cu1—N1	93.11 (16)
O1^i^—Cu1—O1	95.6 (2)
N1^i^—Cu1—N1	95.7 (2)

Symmetry code: (i) 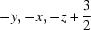.

**Table 2:** Hydrogen-bond geometry (Å, °) *Cg*1, *Cg*2 and *Cg*3 are the centroids of the Cu/O1/C1/C6/C7/N1, Cu/O1′/C1′/C6′/C7′/N1′ and C1–C6 rings, respectively.

*D*—H⋯*A*	*D*—H	H⋯*A*	*D*⋯*A*	*D*—H⋯*A*
C3—H3⋯*Cg*1^ii^	0.93	2.85	3.415 (6)	120
C3—H3⋯*Cg*2^iii^	0.93	2.85	3.415 (6)	120
C12—H12*A*⋯*Cg*3^iv^	0.93	2.76	3.458 (6)	132

Symmetry codes: (ii) 
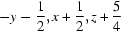
; (iii) 
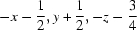
; (iv) 
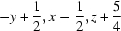
.

**Fig. 1.**
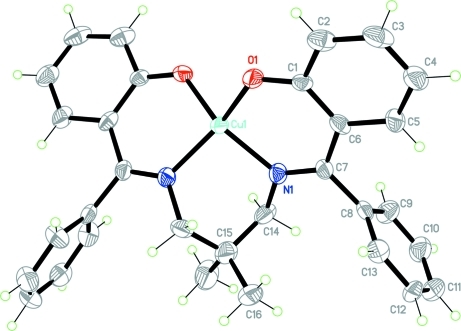
The molecular structure of the title compound, showing 30% probability displacement ellipsoids and the atomic numbering.

## supplementary crystallographic information

### Comment

Schiff base ligands are one of the most prevalent systems in coordination chemistry. As part of a general study of potentially tetradentate Schiff bases and their complexes (Kargar *et al.*, 2009; Kargar *et al.*, 2010), we have determined the crystal structure of the title compound.

The asymmetric unit of the title compound, Fig. 1, comprises half of the Schiff base complex as the molecule has crystallographically imposed 2-fold symmetry . The geometry around the Cu^II^ atom is distorted square planar, Table 1. The dihedral angle between the two phenyl rings is 76.0 (3)°. The crystal packing is stabilized by the intermolecular C—H···π interactions, Table 2.

### Experimental

The title compound was synthesized by adding an methanolic solution (25 ml) of bis(2-hydroxybenzophenone)-2,2'-dimethyl propanediimine (2 mmol) to a solution of CuCl_2_.4H_2_O (2 mmol in 25 ml ethanol). The mixture was refluxed with stirring for half an hour. The resultant green solution was filtered. Dark-green single crystals for X-ray structure determination were recrystallized from ethanol by slow evaporation of the solvents at room temperature over several days.

### Refinement

The C-bound H atoms were geometrically placed (C–H = 0.93–0.97 Å) and refined as riding with *U*_iso_(H) = 1.2–1.5*U*_eq_(C).

### Crystal data

**Table:**

[Cu(C_31_H_28_N_2_O_2_)]	*D*_x_ = 1.426 Mg m^−^^3^
*M**_r_* = 524.09	Mo *K*α radiation, λ = 0.71073 Å
Tetragonal, *P*4_1_2_1_2	Cell parameters from 374 reflections
Hall symbol: P 4abw 2nw	θ = 2.2–24.9°
*a* = 9.7435 (14) Å	µ = 0.93 mm^−^^1^
*c* = 25.717 (6) Å	*T* = 291 K
*V* = 2441.5 (8) Å^3^	Block, dark-green
*Z* = 4	0.21 × 0.11 × 0.08 mm
*F*(000) = 1092	

### Data collection

**Table:**

STOE IPDS 2T Image Plate diffractometer	2376 independent reflections
Radiation source: fine-focus sealed tube	1380 reflections with *I* > 2σ(*I*)
graphite	*R*_int_ = 0.088
Detector resolution: 0.15 mm pixels mm^-1^	θ_max_ = 26.0°, θ_min_ = 2.2°
ω scans	*h* = −10→3
Absorption correction: multi-scan [*MULABS* (Blessing, 1995) in *PLATON* (Spek, 2009)]'	*k* = −11→12
*T*_min_ = 0.995, *T*_max_ = 1.000	*l* = −27→31
5595 measured reflections	

### Refinement

**Table:**

Refinement on *F*^2^	Secondary atom site location: difference Fourier map
Least-squares matrix: full	Hydrogen site location: inferred from neighbouring sites
*R*[*F*^2^ > 2σ(*F*^2^)] = 0.070	H-atom parameters constrained
*wR*(*F*^2^) = 0.064	*w* = 1/[σ^2^(*F*_o_^2^) + (0.0005*P*)^2^] where *P* = (*F*_o_^2^ + 2*F*_c_^2^)/3
*S* = 0.84	(Δ/σ)_max_ < 0.001
2376 reflections	Δρ_max_ = 0.83 e Å^−^^3^
165 parameters	Δρ_min_ = −0.47 e Å^−^^3^
0 restraints	Absolute structure: Flack (1983), 918 Friedel pairs
Primary atom site location: structure-invariant direct methods	Flack parameter: 0.00 (3)

### Special details

**Table:**

Geometry. All e.s.d.'s (except the e.s.d. in the dihedral angle between two l.s. planes) are estimated using the full covariance matrix. The cell e.s.d.'s are taken into account individually in the estimation of e.s.d.'s in distances, angles and torsion angles; correlations between e.s.d.'s in cell parameters are only used when they are defined by crystal symmetry. An approximate (isotropic) treatment of cell e.s.d.'s is used for estimating e.s.d.'s involving l.s. planes.
Refinement. Refinement of *F*^2^ against ALL reflections. The weighted *R*-factor *wR* and goodness of fit *S* are based on *F*^2^, conventional *R*-factors *R* are based on *F*, with *F* set to zero for negative *F*^2^. The threshold expression of *F*^2^ > σ(*F*^2^) is used only for calculating *R*-factors(gt) *etc*. and is not relevant to the choice of reflections for refinement. *R*-factors based on *F*^2^ are statistically about twice as large as those based on *F*, and *R*- factors based on ALL data will be even larger.

### Fractional atomic coordinates and isotropic or equivalent isotropic displacement parameters (Å^2^)

**Table:**

	*x*	*y*	*z*	*U*_iso_*/*U*_eq_	
Cu1	−0.17381 (7)	0.17381 (7)	0.7500	0.0303 (2)	
O1	−0.1851 (4)	0.3469 (4)	0.71702 (12)	0.0358 (9)	
N1	−0.0615 (4)	0.0946 (4)	0.69403 (16)	0.0277 (11)	
C1	−0.1327 (5)	0.3836 (5)	0.6724 (2)	0.0241 (14)	
C2	−0.1492 (6)	0.5209 (6)	0.6563 (2)	0.0368 (16)	
H2	−0.1866	0.5835	0.6797	0.044*	
C3	−0.1129 (5)	0.5662 (6)	0.6081 (3)	0.0409 (17)	
H3	−0.1289	0.6570	0.5987	0.049*	
C4	−0.0523 (6)	0.4770 (6)	0.5734 (2)	0.0442 (18)	
H4	−0.0287	0.5068	0.5402	0.053*	
C5	−0.0271 (6)	0.3441 (7)	0.5881 (2)	0.0359 (16)	
H5	0.0148	0.2852	0.5644	0.043*	
C6	−0.0622 (5)	0.2935 (5)	0.6374 (2)	0.0253 (13)	
C7	−0.0273 (5)	0.1505 (6)	0.6502 (2)	0.0255 (13)	
C8	0.0569 (6)	0.0710 (6)	0.61152 (19)	0.0285 (13)	
C9	0.0001 (6)	−0.0316 (6)	0.5810 (2)	0.0365 (17)	
H9	−0.0939	−0.0476	0.5828	0.044*	
C10	0.0788 (7)	−0.1105 (7)	0.5483 (2)	0.0459 (19)	
H10	0.0380	−0.1793	0.5286	0.055*	
C11	0.2187 (7)	−0.0879 (7)	0.5445 (3)	0.0479 (19)	
H11	0.2731	−0.1408	0.5226	0.058*	
C12	0.2746 (6)	0.0156 (7)	0.5742 (2)	0.048 (2)	
H12A	0.3684	0.0325	0.5720	0.058*	
C13	0.1964 (5)	0.0942 (5)	0.6069 (2)	0.0352 (16)	
H13	0.2374	0.1639	0.6261	0.042*	
C14	−0.0324 (6)	−0.0503 (5)	0.70648 (18)	0.0356 (15)	
H14A	0.0016	−0.0953	0.6754	0.043*	
H14B	−0.1176	−0.0948	0.7162	0.043*	
C15	0.0712 (6)	−0.0712 (6)	0.7500	0.046 (2)	
C16	0.2175 (5)	−0.0535 (7)	0.7303 (2)	0.069 (2)	
H16A	0.2807	−0.0680	0.7584	0.104*	
H16B	0.2289	0.0377	0.7169	0.104*	
H16C	0.2351	−0.1190	0.7033	0.104*	

### Atomic displacement parameters (Å^2^)

**Table:**

	*U*^11^	*U*^22^	*U*^33^	*U*^12^	*U*^13^	*U*^23^
Cu1	0.0317 (3)	0.0317 (3)	0.0277 (5)	0.0088 (6)	0.0062 (4)	0.0062 (4)
O1	0.044 (2)	0.034 (2)	0.029 (2)	0.013 (3)	0.012 (2)	0.005 (2)
N1	0.030 (3)	0.026 (3)	0.027 (3)	0.001 (2)	−0.001 (2)	0.005 (2)
C1	0.016 (3)	0.028 (3)	0.028 (3)	0.002 (3)	0.000 (3)	0.006 (3)
C2	0.027 (4)	0.033 (4)	0.050 (4)	0.002 (3)	0.000 (4)	0.007 (3)
C3	0.030 (4)	0.033 (4)	0.060 (5)	0.002 (3)	0.003 (4)	0.021 (4)
C4	0.043 (4)	0.047 (4)	0.042 (4)	0.006 (4)	0.005 (4)	0.021 (4)
C5	0.033 (4)	0.043 (4)	0.032 (4)	−0.005 (4)	0.005 (3)	0.008 (4)
C6	0.021 (3)	0.028 (4)	0.027 (3)	−0.001 (3)	−0.002 (3)	0.009 (3)
C7	0.020 (3)	0.036 (4)	0.021 (3)	−0.001 (3)	−0.006 (3)	0.001 (3)
C8	0.034 (4)	0.032 (4)	0.019 (3)	0.000 (3)	−0.007 (3)	0.010 (3)
C9	0.032 (4)	0.037 (4)	0.041 (4)	0.002 (3)	0.000 (3)	−0.006 (3)
C10	0.061 (5)	0.044 (5)	0.032 (4)	0.008 (4)	0.002 (4)	−0.007 (3)
C11	0.050 (5)	0.058 (5)	0.035 (4)	0.022 (4)	0.010 (4)	−0.008 (4)
C12	0.024 (4)	0.076 (5)	0.044 (5)	0.010 (4)	0.014 (3)	0.020 (4)
C13	0.024 (4)	0.040 (4)	0.042 (4)	−0.011 (3)	0.000 (3)	0.004 (3)
C14	0.044 (4)	0.024 (3)	0.038 (4)	0.005 (3)	0.019 (3)	0.000 (3)
C15	0.048 (3)	0.048 (3)	0.040 (5)	0.018 (5)	0.023 (4)	0.023 (4)
C16	0.048 (5)	0.113 (6)	0.047 (5)	0.037 (4)	0.020 (3)	0.028 (4)

### Geometric parameters (Å, °)

**Table:**

Cu1—O1^i^	1.891 (3)	C8—C9	1.386 (7)
Cu1—O1	1.891 (3)	C9—C10	1.373 (7)
Cu1—N1^i^	1.966 (4)	C9—H9	0.9300
Cu1—N1	1.966 (4)	C10—C11	1.384 (7)
O1—C1	1.305 (5)	C10—H10	0.9300
N1—C7	1.295 (6)	C11—C12	1.377 (8)
N1—C14	1.475 (6)	C11—H11	0.9300
C1—C2	1.410 (7)	C12—C13	1.369 (7)
C1—C6	1.433 (7)	C12—H12A	0.9300
C2—C3	1.362 (7)	C13—H13	0.9300
C2—H2	0.9300	C14—C15	1.521 (6)
C3—C4	1.380 (8)	C14—H14A	0.9700
C3—H3	0.9300	C14—H14B	0.9700
C4—C5	1.371 (8)	C15—C14^i^	1.521 (6)
C4—H4	0.9300	C15—C16^i^	1.522 (6)
C5—C6	1.405 (6)	C15—C16	1.522 (6)
C5—H5	0.9300	C16—H16A	0.9600
C6—C7	1.471 (7)	C16—H16B	0.9600
C7—C8	1.504 (7)	C16—H16C	0.9600
C8—C13	1.383 (7)		
			
O1—Cu1—N1	93.11 (16)	C10—C9—C8	121.8 (6)
O1^i^—Cu1—O1	95.6 (2)	C10—C9—H9	119.1
O1^i^—Cu1—N1^i^	93.11 (16)	C8—C9—H9	119.1
O1—Cu1—N1^i^	147.96 (16)	C9—C10—C11	120.3 (7)
O1^i^—Cu1—N1	147.96 (16)	C9—C10—H10	119.9
N1^i^—Cu1—N1	95.7 (2)	C11—C10—H10	119.9
C1—O1—Cu1	128.0 (3)	C12—C11—C10	117.8 (6)
C7—N1—C14	122.8 (4)	C12—C11—H11	121.1
C7—N1—Cu1	127.9 (4)	C10—C11—H11	121.1
C14—N1—Cu1	108.9 (3)	C13—C12—C11	122.0 (6)
O1—C1—C2	118.3 (5)	C13—C12—H12A	119.0
O1—C1—C6	124.9 (5)	C11—C12—H12A	119.0
C2—C1—C6	116.8 (5)	C12—C13—C8	120.6 (6)
C3—C2—C1	123.1 (6)	C12—C13—H13	119.7
C3—C2—H2	118.5	C8—C13—H13	119.7
C1—C2—H2	118.5	N1—C14—C15	114.6 (4)
C2—C3—C4	119.8 (6)	N1—C14—H14A	108.6
C2—C3—H3	120.1	C15—C14—H14A	108.6
C4—C3—H3	120.1	N1—C14—H14B	108.6
C5—C4—C3	119.6 (6)	C15—C14—H14B	108.6
C5—C4—H4	120.2	H14A—C14—H14B	107.6
C3—C4—H4	120.2	C14—C15—C14^i^	111.3 (7)
C4—C5—C6	122.5 (6)	C14—C15—C16^i^	107.1 (3)
C4—C5—H5	118.8	C14^i^—C15—C16^i^	111.2 (3)
C6—C5—H5	118.8	C14—C15—C16	111.2 (3)
C5—C6—C1	118.0 (5)	C14^i^—C15—C16	107.1 (3)
C5—C6—C7	118.6 (5)	C16^i^—C15—C16	108.9 (7)
C1—C6—C7	123.4 (5)	C15—C16—H16A	109.5
N1—C7—C6	122.2 (5)	C15—C16—H16B	109.5
N1—C7—C8	119.9 (5)	H16A—C16—H16B	109.5
C6—C7—C8	117.8 (5)	C15—C16—H16C	109.5
C13—C8—C9	117.4 (5)	H16A—C16—H16C	109.5
C13—C8—C7	120.6 (5)	H16B—C16—H16C	109.5
C9—C8—C7	121.9 (5)		
			
O1^i^—Cu1—O1—C1	−148.3 (5)	C14—N1—C7—C8	−5.7 (8)
N1^i^—Cu1—O1—C1	106.7 (5)	Cu1—N1—C7—C8	−178.3 (3)
N1—Cu1—O1—C1	0.9 (5)	C5—C6—C7—N1	−176.9 (5)
O1^i^—Cu1—N1—C7	100.2 (5)	C1—C6—C7—N1	1.7 (8)
O1—Cu1—N1—C7	−5.5 (5)	C5—C6—C7—C8	6.1 (7)
N1^i^—Cu1—N1—C7	−154.7 (5)	C1—C6—C7—C8	−175.2 (4)
O1^i^—Cu1—N1—C14	−73.3 (5)	N1—C7—C8—C13	−101.0 (6)
O1—Cu1—N1—C14	−178.9 (3)	C6—C7—C8—C13	76.0 (6)
N1^i^—Cu1—N1—C14	31.9 (3)	N1—C7—C8—C9	76.0 (7)
Cu1—O1—C1—C2	−177.3 (4)	C6—C7—C8—C9	−107.0 (6)
Cu1—O1—C1—C6	4.5 (8)	C13—C8—C9—C10	1.7 (8)
O1—C1—C2—C3	−172.2 (5)	C7—C8—C9—C10	−175.5 (6)
C6—C1—C2—C3	6.2 (9)	C8—C9—C10—C11	−0.7 (10)
C1—C2—C3—C4	−2.5 (9)	C9—C10—C11—C12	−0.3 (10)
C2—C3—C4—C5	−1.1 (9)	C10—C11—C12—C13	0.3 (10)
C3—C4—C5—C6	0.8 (9)	C11—C12—C13—C8	0.7 (9)
C4—C5—C6—C1	3.0 (8)	C9—C8—C13—C12	−1.7 (8)
C4—C5—C6—C7	−178.2 (5)	C7—C8—C13—C12	175.5 (5)
O1—C1—C6—C5	172.0 (5)	C7—N1—C14—C15	113.2 (5)
C2—C1—C6—C5	−6.2 (7)	Cu1—N1—C14—C15	−73.0 (5)
O1—C1—C6—C7	−6.7 (8)	N1—C14—C15—C14^i^	39.7 (3)
C2—C1—C6—C7	175.1 (5)	N1—C14—C15—C16^i^	161.4 (5)
C14—N1—C7—C6	177.4 (4)	N1—C14—C15—C16	−79.7 (7)
Cu1—N1—C7—C6	4.8 (8)		

Symmetry codes: (i) −*y*, −*x*, −*z*+3/2.

### Hydrogen-bond geometry (Å, °)

**Table:**

Cg1, Cg2 and Cg3 are the centroids of the Cu/O1/C1/C6/C7/N1, Cu/O1'/C1'/C6'/C7'/N1' and C1–C6 rings, respectively.

**Table:**

*D*—H···*A*	*D*—H	H···*A*	*D*···*A*	*D*—H···*A*
C3—H3···*Cg*1^ii^	0.93	2.85	3.415 (6)	120
C3—H3···*Cg*2^iii^	0.93	2.85	3.415 (6)	120
C12—H12A···*Cg*3^iv^	0.93	2.76	3.458 (6)	132

Symmetry codes: (ii) −*y*−1/2, *x*+1/2, *z*+5/4; (iii) −*x*−1/2, *y*+1/2, −*z*−3/4; (iv) −*y*+1/2, *x*−1/2, *z*+5/4.
